# Depositional Environment of Mio-Pliocene Siwalik Sedimentary Strata from the Darjeeling Himalayan Foothills, India: A Palynological Approach

**DOI:** 10.1371/journal.pone.0150168

**Published:** 2016-03-01

**Authors:** Sandip More, Dipak Kumar Paruya, Suchana Taral, Tapan Chakraborty, Subir Bera

**Affiliations:** 1 Centre of Advanced Study, Department of Botany, University of Calcutta, 35, Ballygunge Circular Road, Kolkata, 700019, West Bengal, India; 2 Geological Studies Unit, Indian Statistical Institute, 203, B.T. Road, Kolkata, 700108, West Bengal, India; Institute of Botany, CHINA

## Abstract

A rich and diverse palynoassemblage recovered from the Churanthi River section (26°53' 59.3" N, 88°34' 17.2" E), Darjeeling foothills Eastern Himalaya, has yielded 87 species assigned to 69 genera. The palynoassemblage is rich in angiosperm taxa (45.63%) followed by gymnosperms (0.45%), pteridophytes (18.49%) and fungal remains (23.88%). Based on their nearest living relatives, a wet evergreen to semi-evergreen forest under a humid tropical to sub-tropical environment during the Mio-Pliocene age has been suggested. A lot of angiosperms such as *Palaeosantalaceaepites*, *Araliaceoipollenites*, *Malvacearampollis*, *Zonocostites*, *Neocouperipollis*, *Dicolpopollis*, *Palmidites*, *Palmaepollenites*, isolated salt glands of mangrove plant leaves (*Heliospermopsis*) and *Mediaverrunites* type of fungal spores, along with ichnofossils like *Planolites*, *Palaeophycus*, *Skolithos*, *Rosselia*, *Ophiomorpha* and *Teichichnus* associated with rippled mudstone-siltstone suggest an environment strongly influenced by brackish water. Primary sedimentary structures in the associated strata indicate strong wave agitation common in shallow marine setting. Some high elevation components (5.14%) such as *Alnipollenites*, cf. *Corylus* (Betulaceae), *Juglanspollenites*, *Engelhardtioipollenites* (Juglandaceae), *Quercoides*, *Cupuliferoidaepollenites*, *Lithocarpus*, *Castanopsis* (Fagaceae), *Abietineaepollenites* (Pinaceae) represent hinterland vegetation possibly transported to the prograding deltaic coastline by the rivers. Reworked palynotaxa (*Striatopodocarpites* sp., *Striatites* sp., *Faunipollenites* sp., *Circumstriatites* sp., *Crescentipollenites* sp., *Cuneatisporites* sp., *Parasaccites* sp., *Scheuringipollenites* sp., *Rhizomaspora* sp., *Marsupipollenites* sp., *Lophotriletes* sp.) of Permian age have also been recorded in the palynoassemblage (11.55%) indicating the abundance of Permian Gondwana strata in the source area.

## Introduction

The study of paleovegetation and paleoclimate from the sedimentary rocks of the Siwalik Group through the recovery of micro plant remains is well known from western to central Himalayan sectors [1–11]. Although the Siwalik fossiliferous localities of eastern Himalaya have been proved as important sites recording a variety of mega-fossils [12–20], systematic study of the palynological records for the stratigraphic correlation, paleoclimate reconstruction and depositional setting are scanty [21]. This paper examines paleovegetation pattern from the middle Siwalik deposits exposed in the southern part of the Churanthi River (26°53'59.3" N, 88°34'17.2" E) in the Jalpaiguri District, West Bengal. In this article we combine our data from palynological study with stratigraphic, sedimentologic and ichnofossil data to evolve a clearer understanding of the climatic and depositional setting of the Mio-Pliocene Siwalik rocks of eastern Himalaya [22–24].

## Geological Setting

Siwalik sediments were deposited in the foredeep developed ~ 18 Ma ago along the southern margin of the Himalaya [25–27]. In the far eastern part of the foreland basin (Arunachal Pradesh) the base of the exposed Siwalik sediments has been dated as ~13 Ma [23, 24]. The deposits of the Siwalik succession have traditionally been inferred as representing continent-interior channel-floodplain successions that were part of large megafans, similar to the Kosi megafan in the present-day Gangetic alluvial plain. [28–30].

**Fig 1:**
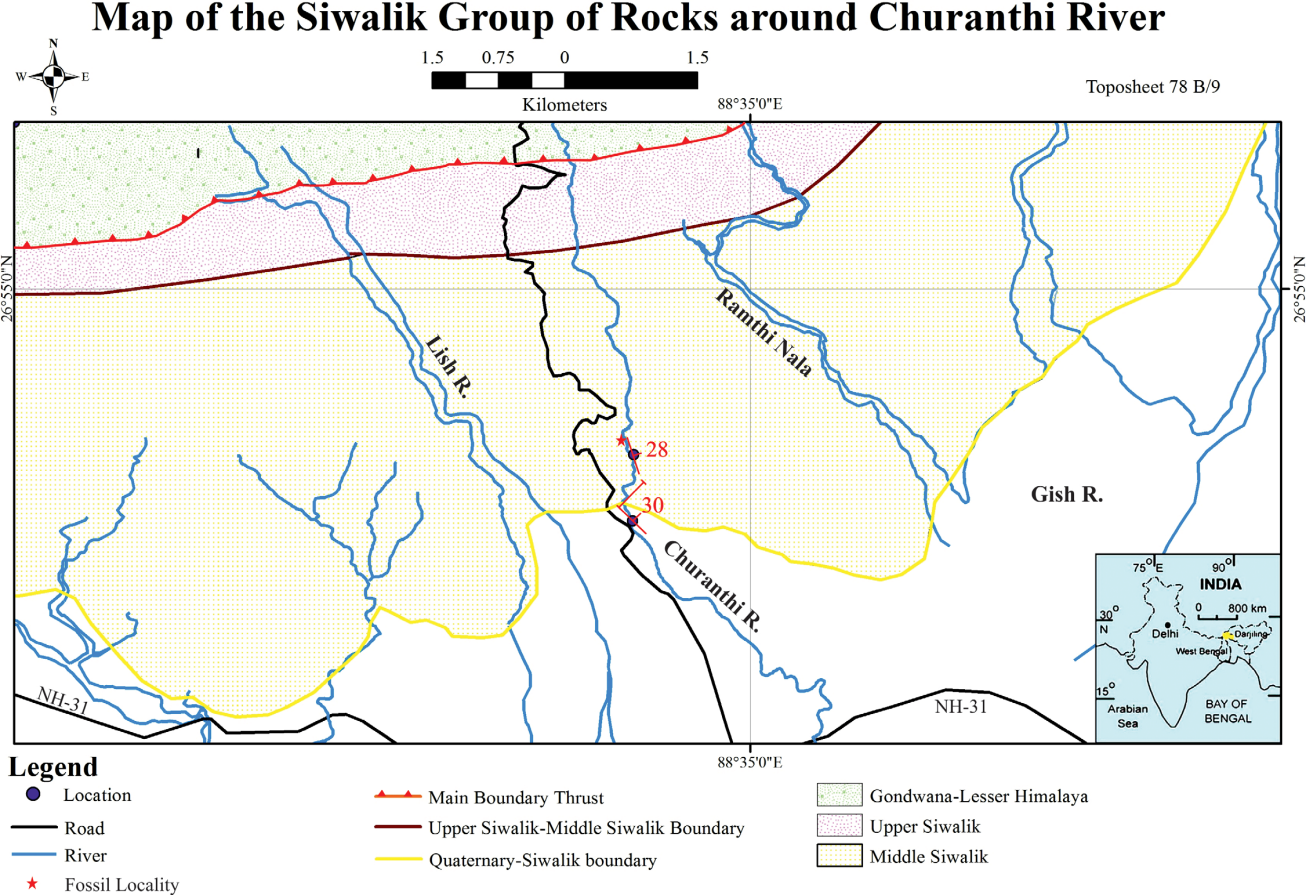
Detailed map of the Siwalik rocks of the study area around Churanthi River, showing the bedding plane orientations and fossil locality. The detailed sedimentological log (Fig 2A) was measured along the red line.

**Fig 2:**
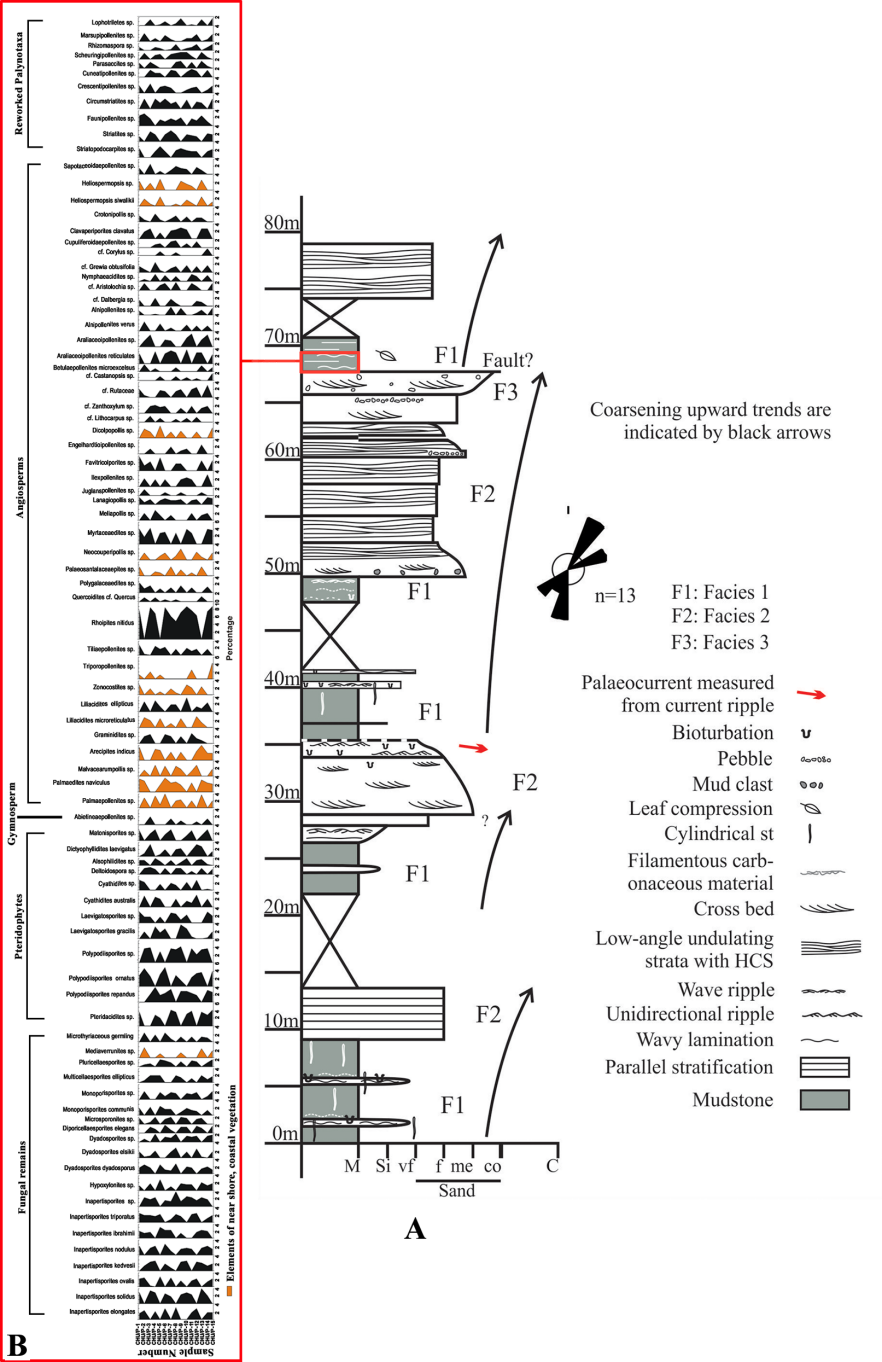
A. Detailed sedimentological log of the Siwalik rocks exposed along the southern part of Churanthi River; B. Pollen diagram showing the percentages of recovered palynotaxa from Churanthi River section of Darjeeling foothills, West Bengal, India.

On the basis of lithology and faunal characters, the Siwalik succession is divided into three broad stratigraphic divisions i.e. Lower (middle Miocene—lower Pliocene), Middle (Pliocene) and Upper Siwalik subgroups (Pliocene- lower Pleistocene) [22, 31–34] although the sediments of the Siwalik in the eastern Himalaya exhibit major differences with those in the type section in the western Himalaya in terms of the facies and faunal assemblage [35]. Acharyya [36] divided the Siwalik succession into lower, middle and upper units, and named them in the stratigraphic order as the Chunabati Formation/Gish Clay, Geabdat Formation and Parbu Grit Formation (Table 1). Fig 1 shows Siwalik strata within the study area and Fig 2A shows the succession from which our palynoassemblage was recovered. Considering the paucity of palynological data from the Siwalik succession of Darjeeling Himalaya, we explore comprehensively the paleovegetational scenario and environmental conditions during Middle Siwalik time through a detailed palynofloral analysis from the Churanthi River section, and combine the analytical results with the data from trace fossils and sedimentology of the studied succession.

**Table 1:** Stratigraphy of the Siwalik succession of Darjeeling foothills modified after Acharyya [36].

Formation	Group	Age
**Damuda Formation**	Damuda	Permian
***Main Boundary Thrust* (MBT)**		
**Chunabati Formation** [contains horses of Damuda (Miocene) and slivers of Early Miocene limestone]	Lower Siwalik	Early Miocene
***South Kalijhora Thrust* (SKT)**		
**Murti Boulder Bed**	Upper Siwalik	Neogene
**Parbu Grit**	Upper Siwalik	Neogene
**Geabdat Sandstone**	Middle Siwalik	Neogene
**Gish Clay**	Lower Siwalik	Neogene
***Main Frontal Thrust* (MFT)**		
Alluvial topped sediment of Ganga-Brahmaputra Basin		

**Fig 3:**
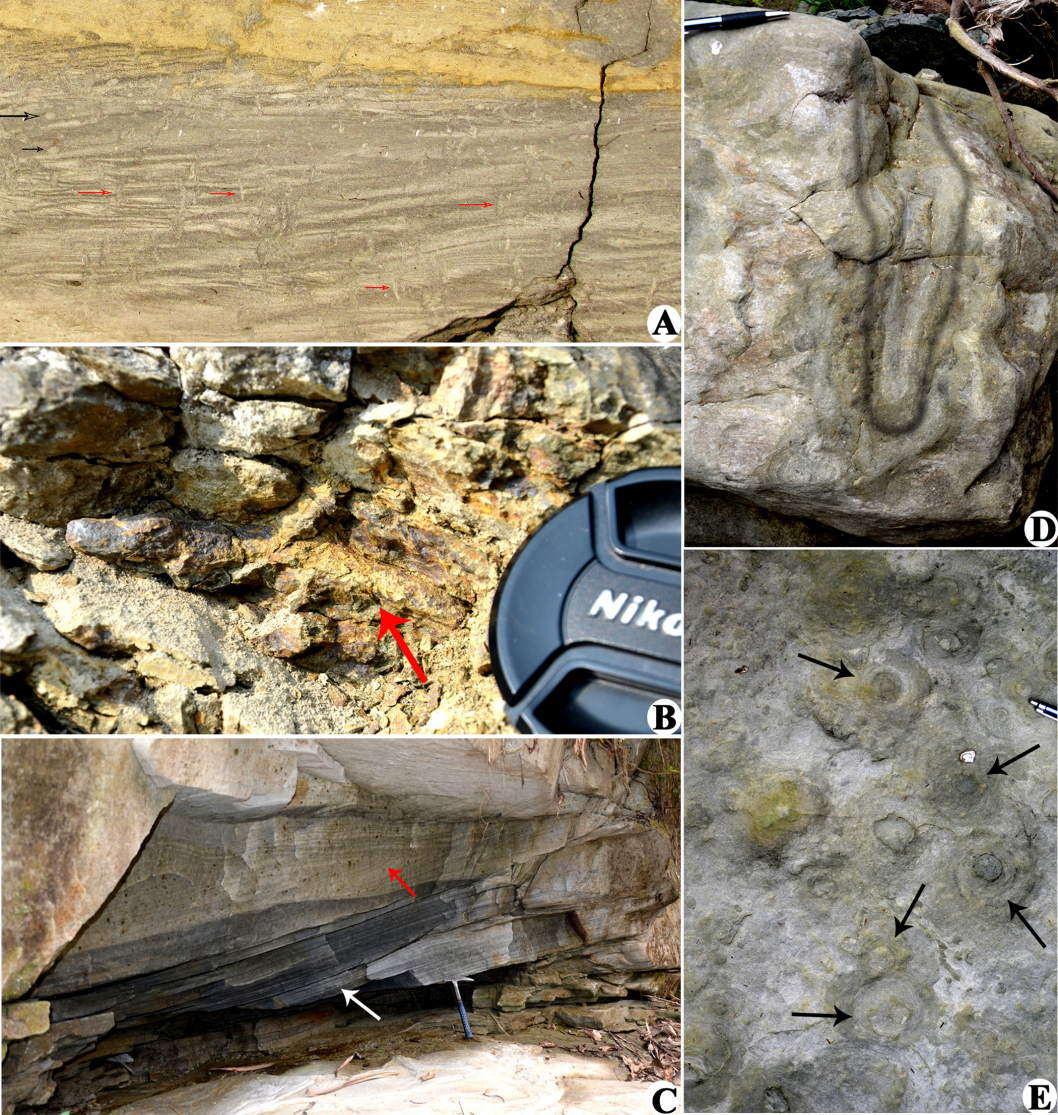
Trace fossils and sedimentary structures from Churanthi River section. A. Bioturbated ripple laminated silt-claystone. (*Planolites* marked by black arrows and *Skolithos* marked by red arrows). B. *Palaeophycus*; C. Low-angled cross stratifications and plane parallel strata; D. *Rosselia*, sectional view: funnel shaped burrow, occurring in very fine grained sandstone (F5), 318 m north of measured section; E. *Rosselia*, bedding plane view: (Note the concentric rings around sandy core).

The Siwalik succession in the southern part of the Churanthi River comprises a sandstone and mudstone succession, the lower 80 m of which is shown in the detailed sedimentological log (Fig 2A). Three major facies are encountered in the logged part of the succession: Facies 1) 5–7 m thick mudstone-siltstone units with interlayered 10–60 cm thick wave-rippled fine-grained sandstone; Facies 2) 3 to 7.5 m thick fine- to medium-grained sandstone with low-angle cross bedding, undulating and plane parallel beds (Fig 3C) and Facies 3) comprising 3–6 m thick cross-stratified coarse grained sandstone with thin pebbly layers in places.

## Materials and Methods

### Samples

Fifteen samples were collected from Churanthi River sections of Mio-Pliocene Siwalik sediments (26°53'59.3" N, 88°34'17.2" E) of the Darjeeling foothills (S1 Table, see also Fig 1). Samples were collected from the grey fine-grained silty clay to chocolate colored clay that dominates the lower part of Middle Siwalik succession and a detailed sedimentological log was prepared including the immediately over- and underlying successions (Fig 2A). Sediments from river traverse sections (each about 30g) were collected in plastic bags for extraction of palynomorphs.

### Palynological methods

Standard maceration techniques based on the methods outlined by Uesugui [37] were adopted for recovery of palynomorphs with some modifications. Each sample (10 gms) was treated with 10% aqueous solution of HCl (hydrochloric acid) to dissolve carbonates (if any), then washed thoroughly with distilled water followed by 40% HF (hydrofluoric acid) treatment for 24 hours. The HF free samples were then treated with conc. HNO_3_ (nitric acid) for a few hours to a few days depending on the degree of maturation of the samples. After thorough washing, samples were treated with 5–10% KOH (potassium hydroxide), sieved (85 size BS-410/69 mesh), washed with the distilled water and dried partially. The samples were then suspended in a heavy liquid of KI (potassium iodide) + CdI_2_ (cadmium iodide) mixture and adjusted to a specific gravity of 2.3. After centrifugation supernatant was retained. To the supernatant, 5 times distilled water was added and then a few drops of 10% glacial acetic acid were added and kept overnight. Finally, permanent slides were prepared using Euperol as mountant for study. Field photographs of fossil exposures documenting the sedimentary structures, the geomorphology of the area and adjacent vegetation were taken using a digital camera (Canon Power Shot SX 120 IS). Photographs of micro plant remains were taken using a transmitted light compound microscope (Zeiss Axioskop 40). Collected samples, residues and slides are kept in the repository of Palaeobotany-Palynology Laboratory, Department of Botany, University of Calcutta, Kolkata, India.

## Results

**Fig 4:**
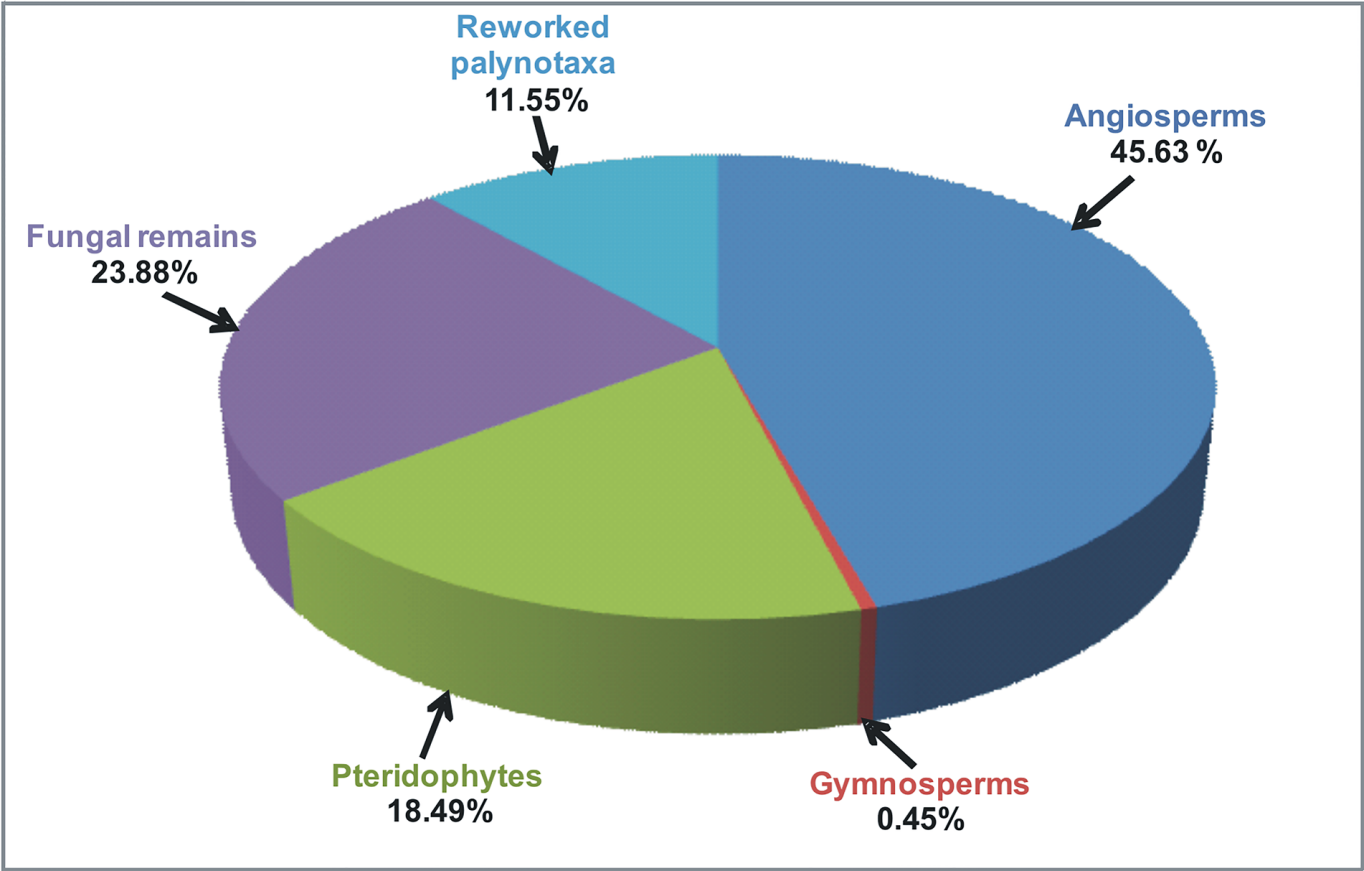
Percentage of palynotaxa in Middle Siwalik sediments of Churanthi River section of Darjeeling foothills.

**Fig 5:**
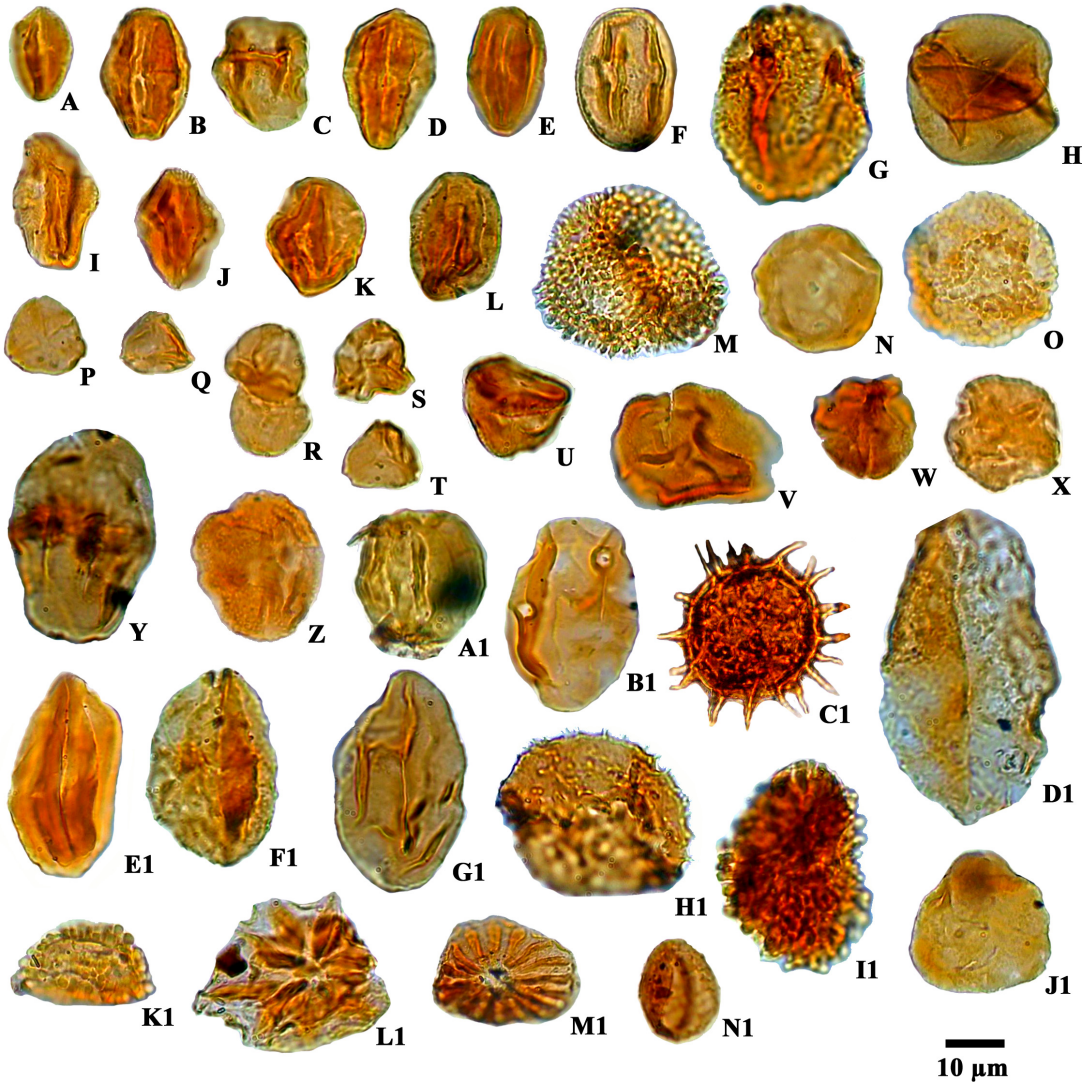
Photomicrographs of angiosperm pollen grains recovered from the Siwalik succession: A. cf. *Lithocarpus* sp.B, D, K. *Rhoipites nitidus*. C. *Zonocostites* sp. E. cf. *Castanopsis* sp. F. cf. *Dalbergia* sp.G. cf. *Grewia obtusifolia*.H. *Graminidites* sp.I, J. *Tiliaepollenites* sp. L. *Quercoidites* cf. *Quercus*.M. *Crotonipollis* sp.N. *Juglanspollenites* sp.O. *Lanagiopollis* sp.P, R, T. *Myrtaceaedites* sp. Q. *Engelhardtioipollenites* sp. S. *Triporopollenites* sp. U. cf. Rutaceae. V. *Favitricolporites* sp. W. cf. *Zanthoxylum* sp. X.*Alnipollenites* sp. Y.*Polygalaceaedites* sp. Z.*Araliaceoipollenites reticulatus*.A1. Unidentified. B1. *Meliapollis* sp.C1. *Malvacearumpollis* sp.D1. *Liliacidites ellipticus*. E1.*Cupuliferoidaepollenites* sp.F1. cf. *Aristolochia* sp.G1.Unidentified. H1. *Nymphaeacidites* sp.I1. *Ilexpollenites* sp.J1. *Betulaepollenites microexcelsus*. K1. *Clavaperiporites clavatus*. L1. *Heliospermopsis* sp.M1. *Heliospermopsis siwalikii*.N1. *Neocouperipollis* sp.

**Fig 6:**
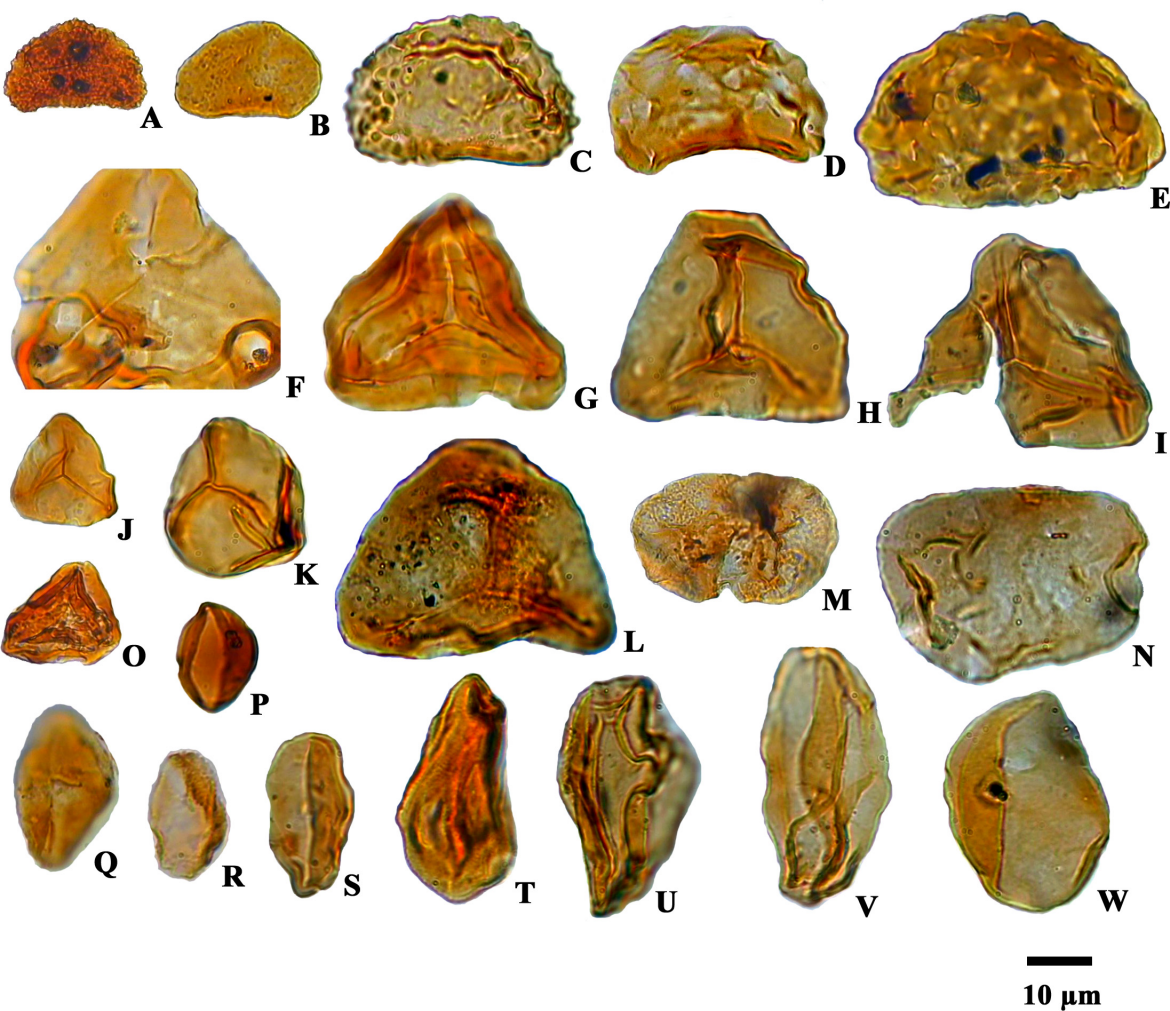
Photomicrographs of pteridophytic spores, gymnosperm and angiosperm pollen grains recovered from the Siwalik succession: A.*Polypodiisporite*s sp. B. *Laevigatosporites gracilis*. C. *Polypodiisporites repandus*. D. *Laevigatosporites* sp. E. Unidentified. F. *Alsophilidites* sp. G, O. *Pteridacidites* sp. H, I. *Cyathidite*s sp. J. *Cyathidites australis*. K. *Dictyophyllidites laevigatus*. L. *Deltoidospora* sp. M. *Abietineaepollenites* sp. N. *Dicolpopollis* sp. P. *Arecipites indicus*. Q. *Palaeosantalaceaepites* sp. R. *Liliacidites microreticulatus*. S, U, V.*Palmaepollenites* sp. T. *Araliaceoipollenites* sp. W. *Palmaedites naviculus*.

**Fig 7:**
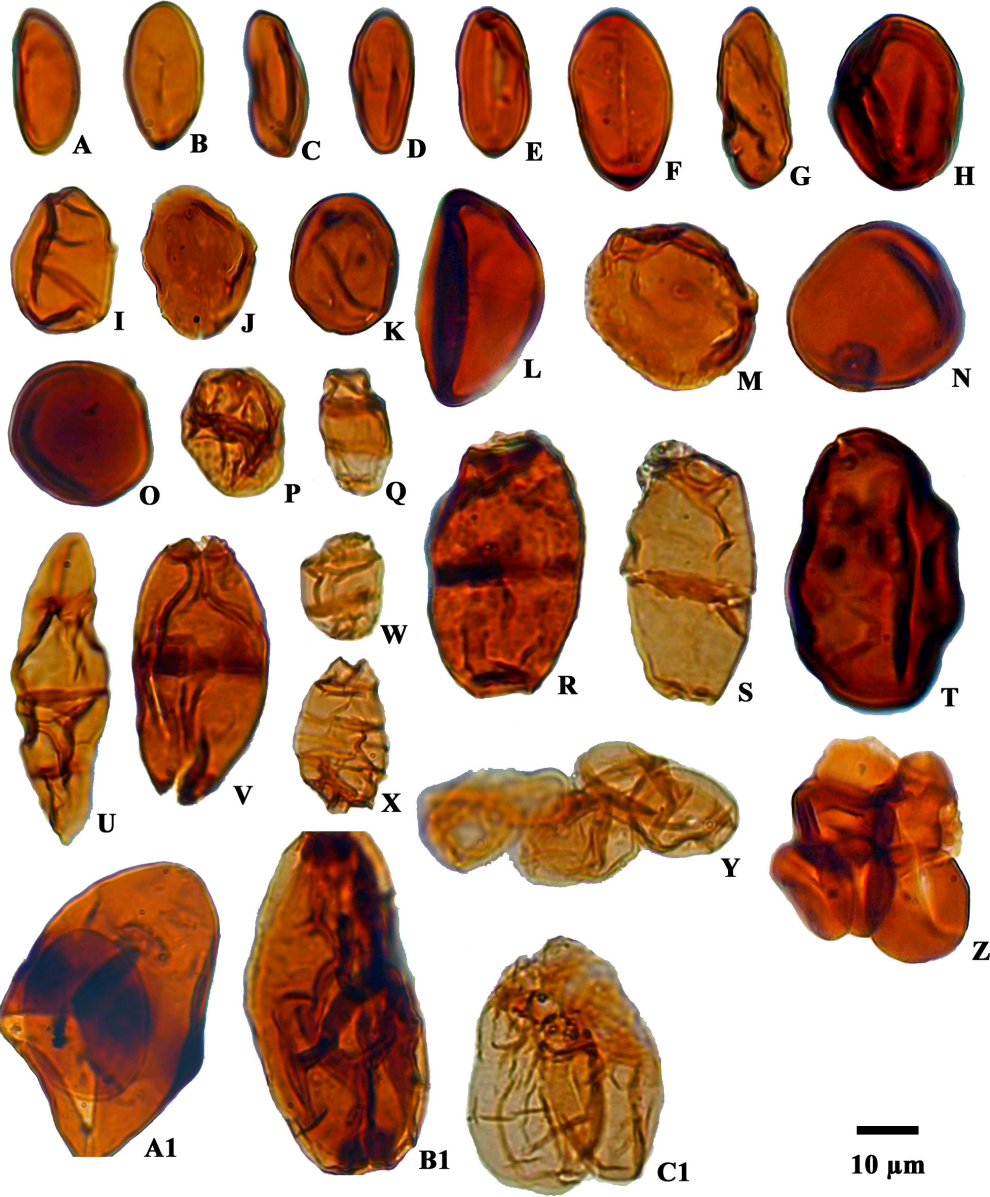
Photomicrographs of fungal spores recovered from the Siwalik succession:A, B, F. *Hypoxylonites* sp.C, G. *Inapertisporites elongates*. D, J, K, L, O, T. *Inapertisporites* sp.E. *Inapertisporites ovalis*.H. *Inapertisporites solidus*. I. *Inapertisporites kedvesii*.M, N. *Inapertisporites nodulus*.P. *Monoporisporites* sp. Q, W, X. *Pluricellaesporites* sp. R. *Dyadosporites dyadosporus*.S. *Dyadosporites* sp.U. *Multicellaesporites ellipticus*. V. *Dyadosporites elsikii*. Y, Z. *Microsporonite*s sp.A1. *Mediaverrunites* sp.B1, C1. Unidentified.

**Fig 8:**
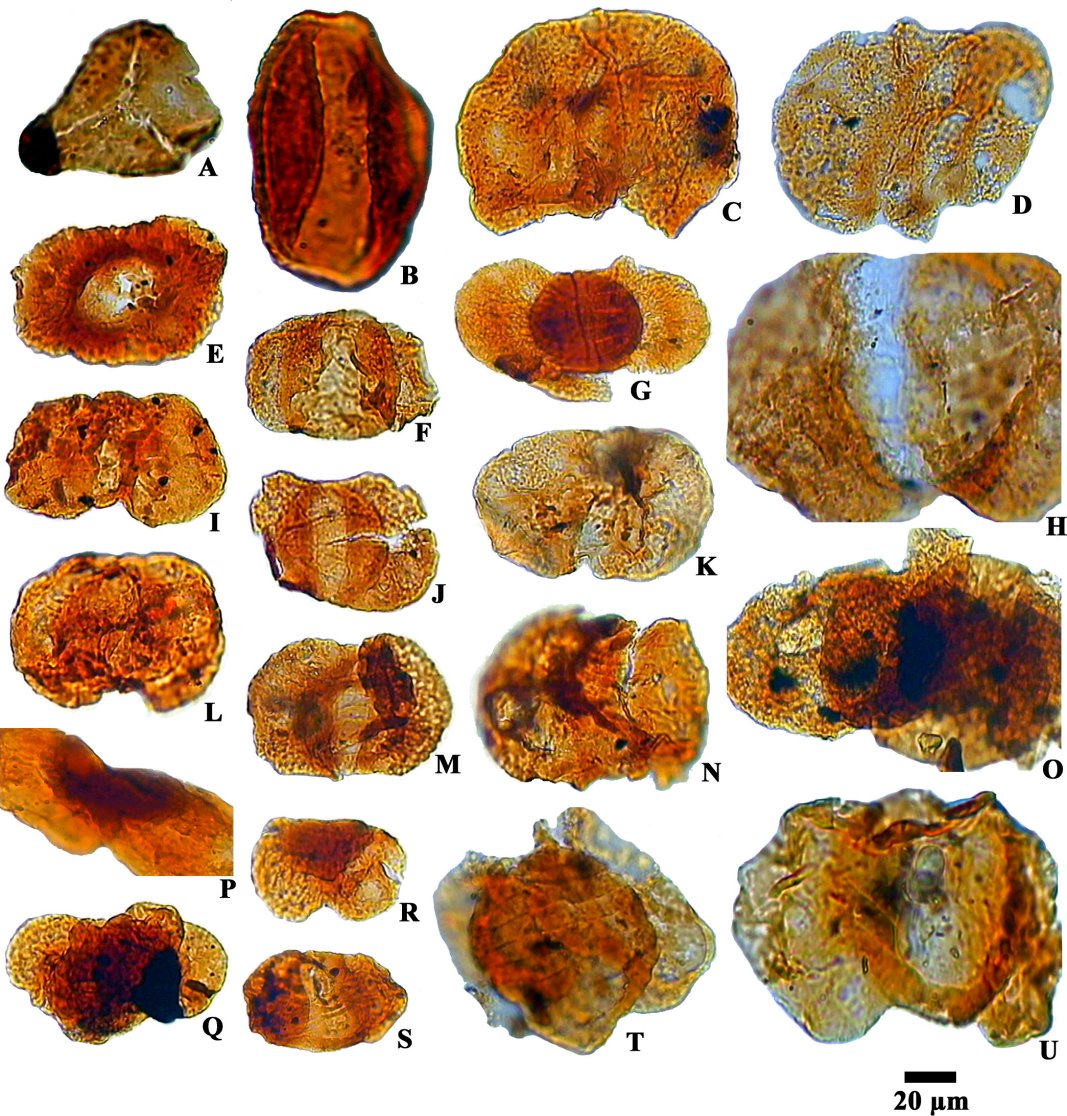
Photomicrographs of reworked palynotaxa of Permian age recovered from the Siwalik succession:A. *Lophotriletes* sp. B. *Marsupipollenites* sp. C, F, I, K, L. *Cuneatipollenites* sp. D. *Scheuringipollenites* sp. E. *Parasaccites* sp. G, R. *Striatites* sp. H, N, Q, T, U. *Striatopodocarpites* sp. J. *Circumstriatites* sp. M. *Crescentipollenites* sp. O. *Rhizomaspora* sp. P. Unidentified. S. *Faunipollenites* sp.

The Siwalik sediments of the Churanthi River section of Darjeeling foothills yielded rich and diverse angiosperms, pteridophytes and fungal remains. In each sample between 100 and 200 palynomorphs (only sample No. CHU/P-5 yielded 87 grains) were counted to determine spore and pollen grain frequency estimates (Fig 2B). The palynoassemblage consisted of 87 species under 69 genera. Angiosperms were numerically most abundant (45.63%) followed by fungi (23.88%), pteridophytes (18.49%) and gymnosperms (0.45%), (Figs 1B and 4). The recovered angiosperm palynotaxa are: *Alnipollenites verus* (Potonié; Banerjee and Nandi 1994), *Alnipollenites* sp., *Araliaceoipollenites reticulatus* (Dutta and Sah, 1974), *Araliaceoipollenites* sp., *Arecipites indicus* (Venkatachala and Rawat, 1972), cf. *Aristolochia* sp., *Betulaepollenites microexcelsus* (Potonié; Kumar and takahashi, 1991), cf. *Castanopsis* sp., *Clavaperiporites clavatus* (Rao and Ramanujam, 1978), cf. *Corylus* sp., *Crotonipollis* sp., *Cupuliferoidaepollenites* sp., cf. *Dalbergia* sp., *Dicolpopollis* sp., *Engelhardtioipollenites* sp., *Favitricolporites* sp., *Graminidites* sp., cf. *Grewia obtusifolia*, *Ilexpollenites* sp., *Juglanspollenites* sp., *Lanagiopollis* sp., *Liliacidites microreticulatus* (Elsik and Dilcher, 1974), *L*. *ellipticus* (Venkatachala and Kar; Kumar, 1994), cf. *Lithocarpus* sp., *Malvacearumpollis* sp., *Meliapollis* sp., *Myrtaceaedites* sp., *Neocouperipollis* sp., *Nymphaeacidites* sp., *Palmaedites naviculus* (Kar and Saxena; Kar and Bhattacharya, 1992), *Palmaepollenites* sp., *Palaeosantalaceaepites* sp., *Polygalaceaedites* sp., *Quercoidites* cf. *Quercus*, *Rhoipites nitidus* (Sah and Dutta, 1968), cf. Rutaceae, *Sapotaceoidaepollenites* sp., *Tiliaepollenites* sp., *Triporopollenites* sp., cf. *Zanthoxylum* sp. and *Zonocostites* sp. along with the isolated salt glands of *Heliospermopsis siwalikii* (Banerjee, 1995) and *Heliospermopsis* sp. (Figs 5 and 6). The only gymnosperm recorded is *Abietineaepollenites* sp., although in very low frequencies (0.45%). *Alsophilidites* sp., *Cyathidites australis* (Couper, 1953), *Cyathidite*s sp., *Deltoidospora* sp., *Dictyophyllidites laevigatus* (Kar, 1985), *Laevigatosporites gracilis* (Wilson and Webster; Naskar and Baksi, 1978), *Laevigatosporites* sp., *Pteridacidites* sp., *Polypodiisporites repandus* (Takahashi; Saxena, 1978), *P*. *ornatus* (Sah; Venkatachala and Rawat, 19731a), *Polypodiisporite*s sp., and *Matonisporites* sp. are found in significant numbers among pteridophytes (Fig 6) and *Dyadosporites dyadosporus* (Salard-Cheboldaeff and Locquin; Kalgutkar & Jansonius, 2000), *D*. *elsikii* (Salard-Cheboldaeff and Locquin; Kalgutkar & Jansonius, 2000), *Dyadosporites* sp., *Diporicellaesporites elegans*, *Hypoxylonites* sp., *Inapertisporites elongatus* (Rouse; Elsik, 1990a), *I*. *solidus* (Songzhichen and Cao Liu 1994), *I*. *ovalis* (Sheffy and Dilcher, 1971), *I*. *kedvesii* (Elsik, 1968), *I*. *nodulus* (Sheffy and Dilcher; Kalgutkar & Jansonius, 2000), *I*. *ibrahimii* (Ediger and Alissan, 1989), *I*. *triporatus* (Rouse, 1962), *Inapertisporites* sp., *Mediaverrunites* sp., *Microsporonite*s sp., *Monoporisporites communis* (Songzhichen, 1985), *Monoporisporites* sp., *Multicellaesporites ellipticus* (Sheffy and Dilcher; Kalgutkar & Jansonius, 2000), *Pluricellaesporites* sp. and a Microthyriaceous germling were retrieved among fungal taxa (Fig 7). A good number of reworked taxa of Permian age (11.55%) such as *Circumstriatites* sp., *Crescentipollenites* sp., *Cuneatipollenites* sp., *Faunipollenites* sp., *Lophotriletes* sp., *Striatopodocarpites* sp., *Striatites* sp., *Scheuringipollenites* sp., *Parasaccites* sp., *Rhizomaspora* sp., *Marsupipollenites* sp. were recovered from the sediments (Fig 8). Some ichnofossils associated with wave rippled mudstone-siltstone (facies 1 of measured section) were also identified namely *Planolites*, *Palaeophycus*, *Rosselia* and *Skolithos* (Fig 3). A pollen diagram was prepared using the TILIA-TILIAGRAPH software [38] representing the percentage frequency (%) of individual palynotaxa in the assemblage (Fig 2B). The possible botanical affinities of recovered pollen grains and spores recognized in these assemblages and their present day distribution are presented in S2 Table.

## Discussion

### Palaeoecology and palaeoclimate

The palynological assemblage recovered from the Churanthi River section of the Darjeeling foothills consists of 87 species belonging to 69 genera representing fungi and vascular plant taxa (Fig 2B). Fungi are represented by spores, hyphae and fruiting bodies (23.88%). Vascular plants are represented by pteridophytic spores (18.49%) and pollen grains of angiosperms (45.63%) while gymnosperms are very poorly represented by single taxon, namely *Abietineaepollenites* (0.45%).

The majority of palynomorphs recovered from these sediments belong to flowering plant families including Arecaceae, Anacardiaceae, Araliaceae, Aristolochiaceae, Alangiaceae, Aquifoliaceae, Betulaceae, Euphorbiaceae, Fagaceae, Fabaceae, Juglandaceae, Liliaceae, Linaceae, Myrtaceae, Meliaceae, Nymphaeaceae, Poaceae, Rutaceae, Rhizophoraceae, Sapotaceae and Tiliaceae along with fern plant families namely Cyatheaceae, Lindsaeaceae, Polypodiaceae, Matoniaceae and Pteridaceae, which have affinities to plants growing in wet evergreen to semi-evergreen forests under tropical to subtropical climates (S2 Table, see also Figs 5 and 6). Diverse types of palm pollen grains e.g. *Palmidites naviculus*, *Palmaepollenites* sp., *Arecipites indicus*, *Neocouperipollis* sp. and *Dicolpopollis* sp. along with *Palaeosantalaceaepites* sp., *Zonocostites* sp. (Rhizophoraceae), *Malvacearumpollis* sp. (Malvaceae), *Araliaceoipollenites* sp. (Araliaceae) and isolated salt glands of mangrove plant leaves (*Heliospermopsis siwalikii and Heliospermopsis* sp.) further demonstrates the presence of brackish water in a nearshore marine environment [39–40] (S2 Table, see also Figs 5 and 6).

Fungal spores are stratigraphically and environmentally very significant [41–47]. Various types of fungal spores that occur in high frequencies (23.88%) are categorized into three different groups, namely amerospores, didymospores and phragmospores respectively, and reflect the presence of necrotrophic fungi decomposing forest floor litter (S2 Table). Records of fossil fungal spores such as *Inapertisporites* and other fungal spores are good indicators of high precipitation [48]. The presence of epiphyllous fungi as microthyriaceous germlings (Microthyriaceae) in the sediments, as well as some earlier records of microthyriaceous fruit bodies associated with fossil leaves [48–50] demonstrate the presence of broad leaved wet evergreen to semi-evergreen tropical forest as they require high humidity coupled with high temperature for their growth.

An ecologically significant fungal taxon *Mediaverrunites* sp. was also recovered (Fig 7). It is an indicator of a warm humid tropical environment and has been recorded from Miocene sediments of Mizoram, a northeastern state of India [51]. The distinctive morphology of fossil form-taxa *Mediaverrunites* links it with the recent ascomycete genus *Potamomyces* [52]. *Mediaverrunites* occurs in recent deposits of tropical deltaic regions [53, 54]. Although records of the fungal form taxon *Mediaverrunites* are confined to riverine, deltaic or marine sediments of tropical origin, its modern counterpart is a pantropical lignicolous freshwater fungus occupying terrestrial habitats in tropical and subtropical regions [51]. The occurrence of *Mediaverrunites* in the Siwalik sediments of Churanthi River thus probably indicates a warm tropical deltaic setting during the time of deposition [55].

The palynological assemblage recovered from the Churanthi River section of the Darjeeling foothills has affinities to plants growing in tropical, subtropical, temperate, cosmopolitan humid and coastal deltaic environments (Table 2). Several ecologic and climatic indicator taxa recovered in the assemblage are shown in Table 2.

**Table 2:** Ecological analysis of the recovered palynofossils.

Ecological Groups	Palynotaxa
**Tropical- subtropical plant complex**	*Alsophilidites*, *Cyathidites*, D*ictyophyllidites*, *Laevigatosporites*, *Matonisporites*, *Polypodiisporites*, *Pteridacidites*, *Araliaceoipollenites*, *Crotonipollis*, *Graminidites*, *Liliacidites*, *Myrtaceidites*, *Nymphaeacidites*, *Polygalaceaedites*, *Rhoipites*, *Tilipollenites*
**Temperate plant complex**	*Abietineaepollenites*, *Cupuliferoipollenites*, *Engelhardtioidites*, *Juglanspollenites*, *Quercoidites*
**Back mangrove, near shore plant complex**	*Heliospermopsis*, *Malvacearumpollis*, *Palaeosantalaceaepites*, *Zonocostites*
**Coastal plant complex**	*Arecipites*, *Dicolpopollis*, *Neocouperipollis*, *Palmaepollenites*, *Palmaedites*

The ecological groups of Churanthi River section area show dominance of tropical-subtropical plant remains over the temperate ones. The ecological groups based on recovered palynotaxa along with high incidence of fungal remains indicate a wet evergreen to semi-evergreen forest of tropical-subtropical humid climate within a near marine deltaic ecological habitat.

The occurrence of temperate angiosperm plant families like Betulaceae (*Alnipollenites verus*, *Alnipollenites* sp., *Triporopollenites* sp., *Betulaepollenites microreticulatus*, *Corylus* sp.), Juglandaceae (*Juglanspollenites* sp., *Engelhardtioipollenites* sp.), Fagaceae (*Cupuliferoidaepollenites* having affinity with modern taxa like *Lithocarpus* sp., *Castanopsis* sp., *Quercus* sp.) and a gymnosperm family like the Pinaceae (*Abitineaepollenites* sp.) suggests the existence of a subtropical-temperate forest in the catchment of the Middle Siwalik succession and the palynomorphs from these Himalayan forests seem to be transported to the coastal area by the river discharge [23, 27].

Rich assemblages of mega plant remains from the same Siwalik sedimentary exposures of Darjeeling sub-Himalayas have also been recorded [56–62]. Earlier, climatic parameters of Middle Siwalik sedimentary strata of Darjeeling Himalaya were quantified using CLAMP (Climate Leaf Analysis Multivariate Program) analysis using a calibration dataset that includes sites from India, southern China and Thailand and high resolution gridded climate data. The CLAMP data suggested a warm humid tropical climate with a distinctive monsoonal signature [17]. The mean annual temperature (MAT) was 25.4°C ± 2.8°C (all uncertainties ± 2 sigma) with warm month mean temperatures (WMMTs) of 27.8°C ± 3.39°C and cold month mean temperatures (CMMTs) of 21.3°C ± 4°C. Precipitation estimates had high uncertainties but suggest a weak monsoon with growing season precipitation (GSP) of 242 ± 92 cm and mean monthly growing season precipitation (MMGSP) of 24.5 ± 8.8 cm [17,63].

### Facies analysis

The meters thick mudstone-dominated units are inferred to have accumulated in a quiet water environment, whereas interlayered thinner wave-rippled fine sandstones indicate sandy incursion during events of increased sediment influx. The undulating and low-angle stratified fine-grained sandstone of facies 2 (F2) represents the hummocky stratification and low-angle combined flow dune cross strata [64,65]. Preserved hummocky cross strata usually denote formation below fair weather wave-base during intense wave agitation in the shallow seas. The cross-stratified, coarse-grained sandstone of facies 3 (F3) were presumably associated with unidirectional current related to the river channels flows. The logged succession shows four coarsening upward packages, each 12–30 m thick in which thick units of facies 1 (F1) mudstone is successively overlain by F2 and F3 units. The base of some of the sandstone units are flat, non-erosional and they gradationally overlie mudstone through an intervening unit of wave ripple fine-grained sandstone/siltstone (Fig 2A) implying their deposition in a submerged condition. The coarsening-up sedimentation units are typical of prograding deltaic succession in which mudstones represent deeper water deposits of distal delta front, low-angle stratified fine-grained sandstone represents shallower part of delta front deposits and trough cross-bedded coarse-grained sandstones were deposited by fluvial discharge from distributary channels [66, 67]. This facies association, coupled with the significant thickness of the mudstone units and the lack of evidences of emergence and/or root traces indicate that F1 mudstone-siltstone was deposited in distal delta front environment [67] rather than in a shallow water floodplain lake or interdistributary bay [68].

Trace fossils, associated with rippled mudstone-siltstone (facies 1) of the measured section, are mainly *Planolites* (Fig 3A), *Palaeophycus* (Fig 3B) and *Skolithos* (Fig 3A). The trace fossils of definitive marine affinity like *Rosselia* (Fig 3D and 3E), *Ophiomorpha*, *Teichichnus* are well-preserved in the F1 mudstone at a stratigraphically higher position above the measured log. The trace fossil assemblage, *Planolites*-*Palaeophycus*-*Skolithos*, is attributed to a wide spectrum of depositional environments from continental fresh water to shallow marine brackish water environment [69, 70]. However, their occurrence within wave affected shallow marine facies (Facies 1 and 2), and their close association with the marine diagnostic traces (e.g., *Rosselia*-*Ophiomorpha*-*Teichichnus*) occurring at a stratigraphically higher level of the same succession, strongly imply their marine affinity.

Records of early Miocene planktonic microforaminifera [36] from the Chunabati Formation of the Darjeeling foothills, 20 kms away from the present area of investigation, further supports a shallow marine environmental condition in the region.

The abundance of wave-generated structures and associated marine trace fossils unequivocally indicate that the succession was formed in an environment strongly influenced by brackish water and wave agitation. A variable palaeocurrent direction with a northeast-ward component (Fig 2A), is consistent with a shallow marine wave-influenced environment, as is also observed in the Gish river section of this area. A significant amount of vegetal matter, along with unidirectional trough cross stratified coarse-grained sandstone in the succession indicates fluvial input in the depositional milieu [71]. Thus combining the evidences of both fluvial and marine processes, it is inferred that the middle Siwalik sediments of this area were laid down in a deltaic environment. Instead of a sharp erosional basal surface marked by lag conglomerate and a fining-upward trend typical of the fluvial sandstone bodies, the flat non-erosive base and abundance of wave ripples of the sandstone in the Churanthi section, argues against deposition of these sandstone units from a sub-aerial channels. These features are consistent with delta-mouth channels and delta frontsand bodies [66, 71].

The presence of recycled palynofossils of lower and upper Gondwana sediments in the younger Tertiary strata is a common phenomenon in India especially in the north-eastern India [72–74]. In our study the occurrence of recycled palynotaxa of Permian age viz., *Striatopodocarpites* sp., *Striatites* sp., *Faunipollenites* sp., *Circumstriatites* sp., *Crescentipollenites* sp., *Cuneatisporites* sp., *Parasaccites* sp., *Scheuringipollenites* sp., *Rhizomaspora* sp., *Marsupipollenites* sp., *Lophotriletes* sp. (Fig 8) suggests that the bulk of the material making up the Neogene sediments in this region were derived mostly from Permian rocks. The good preservation of the recycled microfossils suggests that the source area of deposition was not far from the basin. Because Gondwana rocks are widely exposed in the Eastern Himalayan foothill regions, it is likely that these Permian plant remains came from the Gondwana strata. At several localities along the Himalayan foothills, strips of Lower Gondwana rocks sometimes with coal seams, shales and mudstones are found sandwiched between the Cenozoic rocks and older rocks of Lesser Himalayan Succession [36, 75] and must have been the source of the reworked palynomorphs.

## Conclusions

Three important conclusions stem from the present study.

1. The climate was mainly tropical to subtropical with high precipitation supporting a wet evergreen to semi-evergreen forest.
2. The presence of different types of palms and members of Rhizophoraceae, Malvaceae, Araliaceae and *Mediaverrunites* type fungal spores along with the *in situ* brackish water to shallow marine trace fossils demonstrates that the succession was formed in an environment strongly influenced by brackish water.
3. Some subtropical-temperate angiosperm families like Betulaceae, Juglandaceae, Fagaceae and the gymnosperm family Pinaceae were growing in the hinterland mountainous region of the catchment. The occurrence of reworked Permian palynotaxa in these sediments indicates erosion of Gondwanan sediments during the deposition of the Siwalik succession in the Neogene.

## Supporting Information

S1 Table Lithostratigraphic details of palynological samples collected from Churanthi River section, Darjeeling foothills, West Bengal, India (DOCX)

S2 Table List of recovered palynofossil taxa with their botanical affinities (Nearest Living Relative), environment and habitat (DOCX)
