# Supplementary material for: Depositional Environment of Mio-Pliocene Siwalik Sedimentary Strata from the Darjeeling Himalayan Foothills, India: A Palynological Approach
Source: PLoS One. 2016 Mar 1;11(3):e0150168. doi: 10.1371/journal.pone.0150168 (PMC4773139; doi:10.1371/journal.pone.0150168)
Supplement: S1 Table — (DOCX) [file pone.0150168.s001.docx]

| **Serial No.** | **Catalogued Samples** | **Latitude and Longitude** | **Lithology** | **Age** | **Area** | |
| --- | --- | --- | --- | --- | --- | --- |
| **1** | CHU/P-1 | 26°53' 59.3" N 88°34' 17.2" E | Grey fine-grained silt clay | **Lower part of the middle Siwalik** |  | **Churanthi River section, Darjeeling foothills, West Bengal, India** |
| **2** | CHU/P-2 | 26°53' 59.3" N 88°34' 17.2" E | Grey fine-grained silt clay |  |  |  |
| **3** | CHU/P-3 | 26°53' 59.3" N 88°34' 17.2" E | Clay |  |  |  |
| **4** | CHU/P-4 | 26°53' 59.3" N 88°34' 17.2" E | Grey clay with black streaks |  |  |  |
| **5** | CHU/P-5 | 26°53' 59.3" N 88°34' 17.2" E | Chocolate colored clay |  |  |  |
| **6** | CHU/P-6 | 26°53' 59.3" N 88°34' 17.2" E | Clay |  |  |  |
| **7** | CHU/P-7 | 26°53' 59.3" N 88°34' 17.2" E | Clay |  |  |  |
| **8** | CHU/P-8 | 26°53' 59.3" N 88°34' 17.2" E | Clay |  |  |  |
| **9** | CHU/P-9 | 26°53' 59.3" N 88°34' 17.2" E | Grey clay |  |  |  |
| **10** | CHU/P-10 | 26°53' 59.3" N 88°34' 17.2" E | Grey clay |  |  |  |
| **11** | CHU/P-11 | 26°53' 59.3" N 88°34' 17.2" E | Chocolate colored clay |  |  |  |
| **12** | CHU/P-12 | 26°53' 59.3" N 88°34' 17.2" E | Chocolate colored clay |  |  |  |
| **13** | CHU/P-13 | 26°53' 59.3" N 88°34' 17.2" E | Clay |  |  |  |
| **14** | CHU/P-14 | 26°53' 59.3" N 88°34' 17.2" E | Clay |  |  |  |
| **15** | CHU/P-15 | 26°53' 59.3" N 88°34' 17.2" E | Chocolate colored clay |  |  |  |

**S1-Table. Lithostratigraphic details of palynological samples collected from Churanthi River section, Darjeeling foothills, West Bengal, India**
