# Supplementary material for: Depositional Environment of Mio-Pliocene Siwalik Sedimentary Strata from the Darjeeling Himalayan Foothills, India: A Palynological Approach
Source: PLoS One. 2016 Mar 1;11(3):e0150168. doi: 10.1371/journal.pone.0150168 (PMC4773139; doi:10.1371/journal.pone.0150168)
Supplement: S2 Table — (DOCX) [file pone.0150168.s002.docx]

**S2-Table. List of recovered palynofossil taxa with their botanical affinities (Nearest Living Relative), environment and habitat**

| **Plant groups** | **Taxa** | **Botanical affinity** | **Present day Distribution** | **Preferred Habitat** |
| --- | --- | --- | --- | --- |
| **Fungi** | *Diporicellaesporites* *elegans* | Fungi Imperfecti, Phragmosporae | Tropical, humid | Cosmopolitan |
|  | *Multicellaesporites* *ellipticus* | Fungi Imperfecti, Phragmosporae |  |  |
|  | *Pluricellaesporites* sp. | Fungi Imperfecti, Phragmosporae |  |  |
|  | *Dyadosporites* *dyadosporus* | Fungi Imperfecti, Didymosporae |  |  |
|  | *Dyadosporites* *elsikii* | Fungi Imperfecti, Didymosporae |  |  |
|  | *Dyadosporites* sp**.** | Fungi Imperfecti, Didymosporae |  |  |
|  | *Hypoxylonites* sp**.** | Fungi Imperfecti, Amerosporae |  |  |
|  | *Inapertisporites* *kedvesii* | Fungi Imperfecti**,** Amerosporae |  |  |
|  | *Inapertisporites* *nodulus* | Fungi Imperfecti**,** Amerosporae |  |  |
|  | *Inapertisporites* *elongates* | Fungi Imperfecti**,** Amerosporae |  |  |
|  | *Inapertisporites* *solidus* | Fungi Imperfecti**,** Amerosporae |  |  |
|  | *Inapertisporites ibrahimii* | Fungi Imperfecti**,** Amerosporae |  |  |
|  | *Inapertisporites triporatus* | Fungi Imperfecti**,** Amerosporae |  |  |
|  | *Inapertisporites sp.* | Fungi Imperfecti**,** Amerosporae |  |  |
|  | *Inapertisporites* *ovalis* | Fungi Imperfecti**,** Amerosporae |  |  |
|  | *Microsporonites* sp. | Fungi Imperfecti, Amerosporae |  |  |
|  | *Monoporisporites communis* | Fungi Imperfecti**,** Amerosporae |  |  |
|  | *Monoporisporites* sp. | Fungi Imperfecti, Amerosporae |  |  |
|  | Microthyriaceous germling | Microthyriaceae |  | Epiphyllous on broad leaved tropical forest |
|  | *Mediaverrunites sp.* | *Potamomyces armatisporus* (Ascomycetes) | Tropical, brackish water | Restricted to tropical, brackish water environment |
| **Pteridophytes** | *Cyathidites* *australis* | Cyathea (Cyatheaceae) | Tropical to sub-tropical | Characteristic elements of thick tropical to sub-tropical forest |
|  | *Cyathidites* sp. | Cyathea (Cyatheaceae) |  | Characteristic elements of thick tropical forest |
|  | *Alsophilidites* sp. | Cyatheaceae |  |  |
|  | *Deltoidospora* sp. | Lindsaeaceae |  | Perennial ferns |
|  | *Polypodiisporites* *repandus* | Polypodiaceae |  |  |
|  | *Polypodiisporites* *ornatus* | Polypodiaceae |  |  |
|  | *Polypodiisporites* sp. | Polypodiaceae |  |  |
|  | *Laevigatosporites* *gracilis* | Polypodiaceae |  |  |
|  | *Laevigatosporites* sp. | Polypodiaceae |  |  |
|  | *Pteridacidites* sp. | Pteridaceae |  | Shade loving perennial ferns |
|  | *Matonisporites* sp. | *Matonia* sp. (Matoniaceae) | Cosmopolitan | Sub-aquatic to swampy |
|  | *Dictyophyllidites laevigatus* | Matoniaceae |  |  |
| **Gymnosperm** | *Abietineaepollenites* sp*.* | *Abies* sp. (Pinaceae) | Temperate | Tree element temperate forest |
| **Angiosperms** | *Dicolpopollis* sp. | *Calamus* (Arecaceae) | Tropics of Asia | Spiny climber of tropical rain forest up to 1200mt altitude |
|  | *Rhoipites* *nitidus* | Anacardiaceae | Tropics of Indomalaya | Trees of tropical forest |
|  | *Palaeosantalaceaepites* sp. | *Rhizophora* –*Bruguiera* type | Tropical, mainly old world | Tree of back mangrove vegetation |
|  | *Zonocostites* sp. | Rhizophoraceae |  |  |
|  | *Araliaceoipollenites* *reticulatus* | Araliaceae | Tropical | Elements of montane evergreen vegetation |
|  | *Araliaceoipollenites* sp. |  |  |  |
|  | *Arecipites indicus* | Arecaceae |  | Woody members of evergreen vegetation |
|  | *Ilexpollenites* sp. | *Ilex* sp. (Aquifoliaceae) |  | Component of Tropical forest |
|  | *Sapotaceoidaepollenites* sp. | Sapotaceae |  | Member of evergreen vegetation |
|  | *Crotonipollis* sp. | Euphorbiaceae |  | Elements of open forest |
|  | cf. *Dalbergia* sp. | Favaceae |  | Tree element of tropical forest |
|  | cf. *Aristolochia* sp. | Aristolochiaceae |  |  |
|  | *Heliospermopsis* *siwalikii* | Isolated salt gland of *Aegiceras* sp, |  | Tropical back mangrove |
|  | *Heliospermopsis* sp. |  |  |  |
|  | *Neocouperipollis* sp. | Arecaceae | Tropical to sub-tropical | Elements of rain forest, member of near shore, coastal vegetation |
|  | *Palmidites* *naviculus* |  |  |  |
|  | *Palmaepollenites* sp. |  |  |  |
|  | *Malvacearumpollis* sp. | Malvaceae |  |  |
|  | *Liliacidites* *microreticulatus* | Liliaceae |  | Cosmopolitan |
|  | *Liliacidites* *ellipticus* |  |  |  |
|  | *Meliapollis* sp. | Meliaceae |  | Elements of tropical forest |
|  | *Myrtaceidites* sp. | Myrtaceae |  | Trees of tropical forest |
|  | *Tiliaepollenites* sp. | Tiliaceae |  | Tree elements of tropical to sub-tropical forest |
|  | cf. *Grewia obtusifolia* | Tiliaceae |  |  |
|  | cf. Rutaceae | Rutaceae |  |  |
|  | cf. *Zanthoxylum* sp. | Rutaceae |  |  |
|  | *Polygalaceaedites* sp. | *Polygala* sp., Polygalaceae |  | Elements of evergreen forest |
|  | *Lanagiopollis* sp. | *Alangium barbatum* , Alangiaceae |  |  |
|  | *Clavaperiporites* *clavatus* | Linaceae |  | Tropical to lower hill ranges |
|  | *Nymphaeacidites* sp. | Nymphaeaceae |  | Fresh water element |
|  | cf. *Lithocarpus* sp. | *Lithocarpus* sp. (Fagaceae) | Sub-tropical to temperate | Tree element of sub-tropical forest |
|  | *Alnipollenites* *verus* | Betulaceae |  | Tree elements of sub-tropical to temperate forest |
|  | *Alnipollenites* sp. | Betulaceae |  |  |
|  | *Juglanspollenites* sp. | Juglandaceae |  |  |
|  | cf. *Castanopsis* sp. | *Castanopsis* sp., Fagaceae |  |  |
|  | *Quercoidites* cf. *Quercus* sp. | *Quercus* sp., Fagaceae |  |  |
|  | cf. *Corylus* sp. | *Corylus* sp., Betulaceae |  |  |
|  | *Triporopollenites* sp. | Betulaceae |  |  |
|  | *Engelhardtioipollenites* sp. | *Engelhardtia* sp, Juglandaceae |  | Tropical to lower hill ranges |
|  | *Betulaepollenites microreticulatus* | Betulaceae |  | Tree elements of sub-tropical to temperate forest |
|  | *Cupuliferoidaepollenites sp.* | Fagaceae |  | Elements of sub-tropical to temperate forest |
|  | *Graminidites* sp. | Poaceae | cosmopolitan | Herbs in open forest |
|  | *Favitricolporites* sp. | Uncertain | ------------- | ------------- |
